# The Impact of COVID-19 on Physical Performance and Mental Health—A Retrospective Case Series of Belgian Male Professional Football Players

**DOI:** 10.3389/fspor.2021.803130

**Published:** 2021-12-13

**Authors:** Jente Wagemans, Peter Catteeuw, Jan Vandenhouten, Jordi Jansen, Xander de Corte, Ciesse Ceusters, Dirk Vissers

**Affiliations:** ^1^Department of Rehabilitation Sciences and Physiotherapy, Faculty of Medicine and Health Sciences, University of Antwerp, Antwerp, Belgium; ^2^Royal Antwerp Football Club, Performance and Physiotherapy, Antwerp, Belgium

**Keywords:** football, athletes, mental health, performance, COVID-19

## Abstract

**Rationale:** As every season, physical performance of players of Royal Antwerp FC's first team was regularly tested and mental well-being and mood were assessed during football season 2020–2021. Just like in the general population, several professional athletes were infected by SARS-CoV-2 during that season. COVID-19 is a complex disorder affecting multiple body systems, potentially damaging lungs, affecting the cardiovascular system or causing muscle weakness. Therefore, the impact of COVID-19 on performance was a major concern for the affected football players and their entourage.

**Objectives:** To retrospectively examine the influence of COVID-19 on physical performance and mental health in professional football athletes during the season 2020–2021.

**Methods:** Thirty-three professional athletes (age: 25.37 ± 4.11 years; height: 182.75 ± 7.62 cm; weight: 78.90 ± 8.97 kg) of a Belgian first division football club were assessed weekly during the 2020-2021 season. Weekly assessments comprised strength values of the hamstrings, hip abductors and hip adductors, jump performance, a modified Hooper questionnaire to assess mental status and nose swab PCR tests for COVID-19. Data analysis was performed from 2 weeks prior to COVID-19 contamination up to 8 weeks after the first positive test. *Post-hoc* Bonferroni correction was applied when performing statistical analysis.

**Results:** Eleven players tested positive for COVID-19. Duration of contamination was 13 ± 7 days. There was no statistically significant difference before and after COVID-19 infection for jump performance, and adductor and abductor muscle strength (*p* > 0.05). Functional hamstring strength improved significantly 2 weeks (MD: 41.48; 95%CI: −3.79 to 86.75; *p* = 0.009) and 4 weeks (MD: 34.76; 95%CI: −8.91 to 78.42; *p* = 0.019) after COVID-19, whereas mood (MD: −0.60; 95%CI: −1.46 to 0.26; *p* = 0.041), stress levels (MD: −0.83; 95%CI: −1.85 to 0.20; *p* = 0.020) and total wellness (MD: −2.41; 95%CI: −5.25 to 0.43; *p* = 0.015) showed a significant reduction 8 weeks after confirmed COVID-19.

**Conclusion:** Physical performance varied considerably across outcomes before and 8 weeks after COVID-19 contamination in a sample of first division football players. However, affected football players' overall well-being, stress levels and mood diminished after a positive COVID-19 test.

## Introduction

The COVID-19 pandemic has had a disrupting effect on global society, including the suspension of league games, and various sports events (Radzimiński et al., 2021; Schumacher et al., 2021), such as the 2020 Tokyo Olympics and the 2020 European Cup football (Grazioli et al., 2020). After 2 months of confinement, the German national football association recommended players to perform home-based training programs, followed by small-group training sessions (Meyer et al., 2021). Sequentially, German Bundesliga was the first to resume their competition in May 2020. By now, 2020–2021 seasons are completed and suspended sports events have taken place. Nevertheless, people still get infected by COVID-19 due to spreading via direct contact or respiratory droplets (Streeck et al., 2020). In football, direct between-players contact is unavoidable and players cannot always maintain the recommended safe distance of 1.5 m (Schumacher et al., 2021). Then again, football players often keep a distance of more than 1.5 meters during a game and direct contact moments are rather concise (Knudsen et al., 2020). The main hypothesis for COVID-19 spreading between football players is the excessive breathing with increased amount of droplets as a result of exertion (Buonanno et al., 2020). Hence, sports leagues installed a health and safety protocol to be able to resume their respective competitions: strict hygiene and social distancing rules, and repeated polymerase chain reaction (PCR) testing (Meyer et al., 2021).

The capability to achieve high intensity performances such as sprinting and jumping is considered critical in football (Rampinini et al., 2007; Vigne et al., 2010). A study with professional football players in Denmark showed the importance of eccentric hamstring training on short sprints and countermovement jump (CMJ) performance (Krommes et al., 2017). The Nordic hamstring exercise shows to be a reliable measurement method to evaluate eccentric hamstring strength (Opar et al., 2013). Furthermore, adductor strength is associated with greater high speed running (Roe et al., 2016). A ForceFrame Strength Testing System was developed by Vald Performance (Newstead, Queensland, Australia), and displayed great measurement properties to assess adductor strength (Ryan et al., 2019). Physical profiling is customary in professional athletes to evaluate their resilience to the vast physical demands (Ryan et al., 2019). Additional to physical performance, elite athletes need mental resilience to cope with training loads, performance demands and constant pressure from their environment, such as media, teammates, fans and family (Rice et al., 2016). The COVID-19 pandemic and the subsequent suspension of competitions had a detrimental effect on the mental health of professional football players (Souter et al., 2021). Considering the physical and mental demands on elite athletes, the aim of this study is to examine the influence of COVID-19 on physical performance and mental health in professional football players.

## Methods

### Study Design

This retrospective observational study was conducted during the 2020–2021 season.

### Participants

Thirty-three professional male football players from Royal Antwerp Football Club (RAFC) participated in this study. All players volunteered to participate and gave written consent.

### Procedures

During preseason, all players were measured for biometric and anthropological data. Henceforward, jump performance, mental health and functional strength of the hamstrings, hip adductors and hip abductor muscles were assessed weekly. Furthermore, all players were submitted to a PCR test to test for COVID-19 contamination.

#### Jump Performance

Jump height of the athletes was measured using a counter movement jump (CMJ) test, by using the ForceDecks (Dual Force Plate System) by Vald Performance (Newstead, Queensland, Australia). Players were instructed to start with a quick downward squat movement, rapidly followed by an explosive upward movement, jumping as high as possible off of the ground. After each jump, land with both feet on the ForceDecks plates. The CMJ test was performed three consecutive times with a rest time of ten seconds between each jump. Average jump height of three consecutive maximal jumps was calculated.

#### Muscle Strength

Functional strength of the hamstrings, hip adductors and hip abductors muscles were assessed using Vald Performance devices. The functional hamstring strength was measured using NordBord (Hamstring Testing System) by Vald Performance, the players were asked to perform three maximal Nordic hamstring exercises with a rest period of ten seconds between the different executions. The average of the three executions was measured. The hip adductor and hip abductor strength were measured using the ForceFrame by Vald Performance. Players were instructed to perform a maximal inward squeeze followed instantly with a maximal outward push. This test was executed three consecutive times with a rest time of ten seconds between each execution. The average of the three different executions was calculated. The test was performed in a lying position with extended legs and the sensors at the ankles.

#### Mental Status

**Figure 1:**
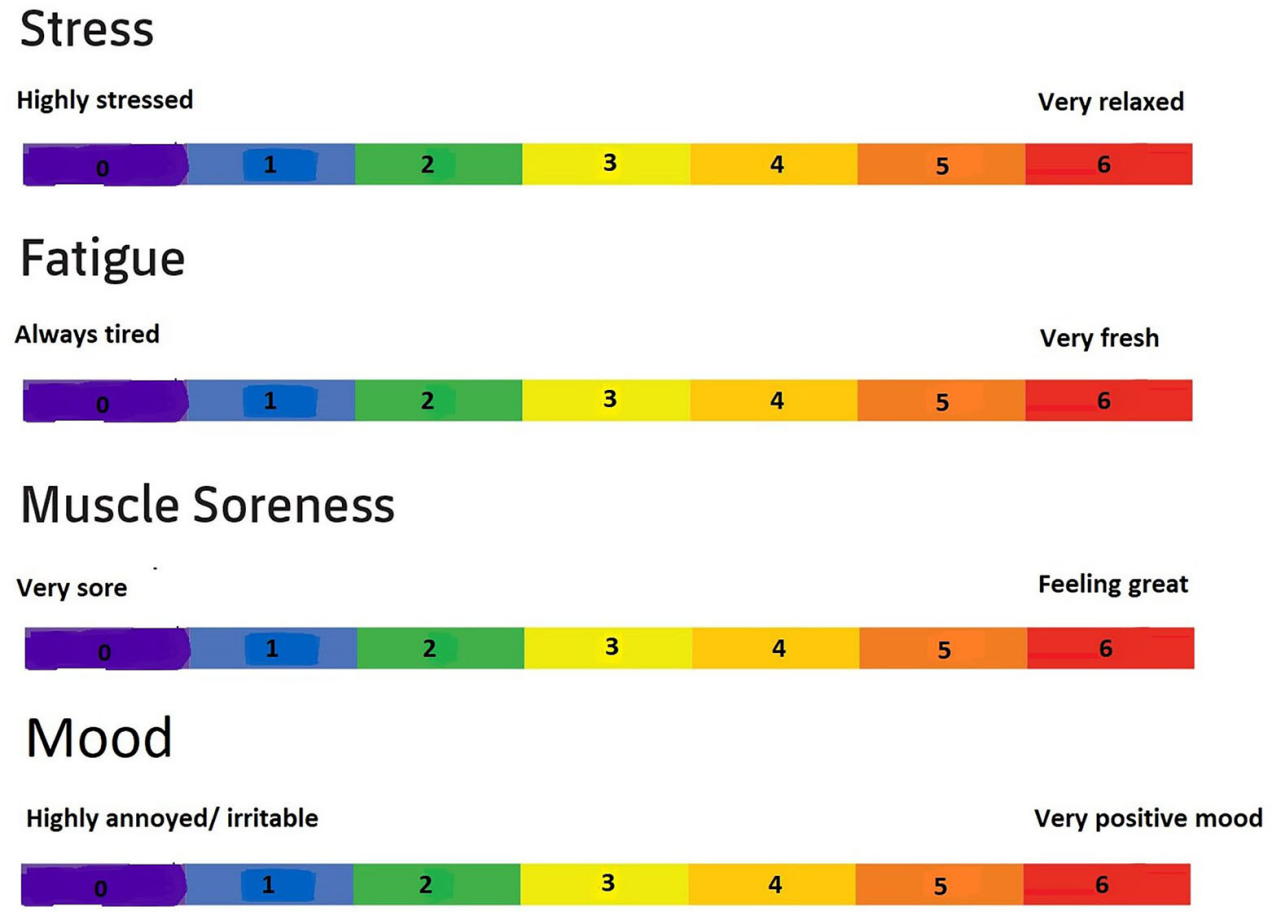
Modified Hooper scale.

Data concerning overall well-being of the players were collected using a questionnaire which was given weekly by the physical staff of the club. This questionnaire was based on the Hooper Scale (Hooper and Mackinnon, 1995). The Hooper scale consists of several subcategories such as fatigue, muscle soreness and stress, that could be scored from 0 to 7. The club's medical staff of modified this scale by adding an extra variable “mood-score” and changing the score range from 0 to 6. The sum of the four variables was also calculated (i.e., the total wellness). The higher the score, the better the players felt. Figure 1 illustrates the modified Hooper scale. Additionally, players reported daily the hours of sleep.

#### COVID-19

COVID−19 infection was examined by using a PCR test, whereby a test sample is collected from the back of the nose by wielding a nose swab (Hadaya et al., 2020).

### Statistical Analysis

All the data were collected in an online database (Smartabase, Fusion Sport, 2011), which was in direct connection with the various devices from Vald Performance. From the online database all previously described data were transferred to Excel and analyzed using IBM SPSS version 27 (IBM company, Armork, New York, USA). Normality and variance of each variable was checked using a Kolmogorov Smirnov test and Levene's test. Linear mixed models were used to see if there were differences between different outcomes pre and post SARS-CoV-2 contamination.

## Results

### Player Profiles

The first team contains 33 players, distinguished by different positions in the field. Table 1 summarizes the players characteristics. During the 2020–2021 season, eleven first-team players were infected with Sars-Cov-2. The positive cases were spread throughout the season. The players who were contaminated played at different positions on the field, namely four wingers, four center-backs and three midfielders were infected.

**Table 1:** Player characteristics.

	***N*** **= 33**
	**Mean ± SD**
Age (years)	25.34 ± 4.61
Height (cm)	182.75 ± 7.42
Weight (kg)	78.90 ± 8.28
BMI	23.33 ± 1.63
Goalkeepers (*n*)	3
Defenders (*n*)	9
Midfielders (*n*)	13
Strikers (*n*)	8
Sars-Cov-2 (*n*)	11
Days unavailable	8.73 ± 4.29
Duration	13 ± 7 days

### Physical Performance

There was no statistically significant difference for countermovement jump height, and adductor and abductor muscle strength after SARS-CoV-2 infection. Players' functional hamstring strength improved significantly 2 weeks (MD: 41.48; 95%CI: −3.79 to 86.75; *p* = 0.009) and 4 weeks (MD: 34.76; 95%CI: −8.91 to 78.42; *p* = 0.019) after the first positive PCR test. However, both results were not statistically significant anymore after Bonferroni correction (2 weeks *p* = 0.087; 4 weeks *p* = 0.193). Table 2 depicts the detailed results of jump performance and muscle strength 1 week prior to COVID-19 infection up to 8 weeks after the first positive test with 2 weeks intervals.

**Table 2:** Physical performance before and after COVID-19.

	**1 week pre COVID**	**2 weeks post COVID**		**4 weeks post COVID**		**6 weeks post COVID**		**8 weeks post COVID**	
	**Mean (SEM)**	**Mean (SEM)**	**MD (95% CI)**	**Mean (SEM)**	**MD (95% CI)**	**Mean (SEM)**	**MD (95% CI)**	**Mean (SEM)**	**MD (95% CI)**
CMJ (cm)	37.41 (1.95)	41.73 (2.58)	4.32 (−4.73 to 13.36)	40.73 (2.29)	3.11 (−4.86 to 11.09)	39.95 (1.79)	2.54 (−3.22 to 8.30)	39.86 (2.14)	2.45 (−4.63 to 9.54)
Hip ABD L (N)	179.03 (9.14)	169.90 (9.76)	−9.13 (−44.03 to 25.76)	161.64 (8.66)	−17.39 (−50.18 to 15.40)	175.27 (9.13)	−3.76 (−37.51 to 29.98)	178.29 (10.51)	−0.74 (−37.79 to 36.31)
Hip ABD R (N)	173.28 (8.02)	161.04 (8.60)	−12.24 (−44.67 to 20.19)	156.39 (7.56)	−16.89 (−47.25 to 13.47)	169.78 (8.01)	−3.50 (−34.76 to 27.76)	175.33 (9.32)	2.05 (−32.29 to 36.39)
Hip ADD L (N)	210.86 (14.52)	196.73 (15.62)	−14.14 (−74.40 to 46.13)	179.06 (13.65)	−31.81 (−88.14 to 24.53)	192.41 (14.51)	−18.46 (−76.50 to 39.58)	200.72 (16.98)	−10.14 (−73.87 to 53.59)
Hip ADD R (N)	212.04 (14.37)	190.72 (15.47)	−21.31 (−82.08 to 39.46)	178.64 (13.48)	−33.40 (−90.12 to 23.32)	196.15 (14.36)	−15.89 (−74.36 to 42.58)	205.11 (16.86)	−6.92 (−71.11 to 57.26)
Nordic L (N)	383.67 (26.41)	409.95 (26.42)	26.28 (−19.61 to 72.16)	421.98 (25.70)	38.32 (−6.10 to 82.65)	397.25 (26.00)	13.58 (−32.02 to 59.18)	390.73 (26.95)	7.07 (−44.70 to 58.84)
Nordic R (N)	404.92 (22.66)	446.40 (22.68)	41.48 (−3.79 to 86.75)*	439.67 (21.85)	34.76 (−8.91 to 78.42)*	428.31 (22.19)	23.40 (−21.51 to 68.29)	421.39 (23.27)	16.47 (−34.54 to 67.48)

*SEM, Standard error of measurement; MD, Mean difference; 95%CI, 95% confidence interval; CMJ, countermovement jump; Hip ABD L, Hip abductor strength left; hip ABD R, Hip abductor strength left; Hip ADD L, Hip adductor strength left; Hip ADD R, Hip adductor strength right; Nordic L, Eccentric Nordic hamstring strength left; Nordic R, Eccentric Nordic hamstring strength right; N, Newton*.

* *p < 0.05*.

### Mental Wellness

Mean results of the modified Hooper scale showed statistically significant reductions in the mood (MD: −0.60; 95%CI: −1.46 to 0.26; *p* = 0.041) and stress level (MD: −0.83; 95%CI: −1.85 to 0.20; *p* = 0.020) subscale scores 8 weeks after confirmed COVID-19. Furthermore, total wellness scores were also significant reduced 8 weeks post COVID-19 (MD: −2.41; 95%CI: −5.25 to 0.43; *p* = 0.015). Conversely, neither results were statistically significantly different after Bonferroni correction (Mood score *p* = 0.409; stress level score *p* = 0.198; total wellness score *p* = 0.148). Detailed results of the modified Hooper scale from 1 week before, up to 8 weeks after the first positive PCR test with 2 weeks intervals are highlighted in Table 3.

**Table 3:** Mental wellness by the modified Hooper Scale before and after COVID-19.

	**1 week pre COVID**	**2 weeks post COVID**		**4 weeks post COVID**		**6 weeks post COVID**		**8 weeks post COVID**	
	**Mean (SEM)**	**Mean (SEM)**	**MD (95% CI)**	**Mean (SEM)**	**MD (95% CI)**	**Mean (SEM)**	**MD (95% CI)**	**Mean (SEM)**	**MD (95% CI)**
Fatigue (0–6)	4.15 (0.37)	4.55 (0.43)	0.93 (−0.80 to 1.58)	4.12 (0.37)	−0.03 (−1.08 to 1.01)	4.13 (0.34)	−0.02 (−0.97 to 0.93)	3.86 (0.33)	−0.30 (−1.22 to 0.62)
Stress level (0–6)	5.32 (0.37)	4.97 (0.44)	−0.35 (−1.67 to 0.99)	5.05 (0.37)	−0.27 (−1.44 to 0.90)	4.54 (0.34)	−0.78 (−1.83 to 0.28)	4.49 (0.33)	−0.83 (−1.85 to 0.20)*
Mood (0–6)	4.85 (0.36)	4.95 (0.41)	0.09 (−1.02 to 1.20)	4.29 (0.36)	−0.57 (−1.55 to 0.41)	4.31 (0.33)	−0.54 (−1.43 to 0.26)	4.26 (0.33)	−0.60 (−1.46 to 0.26)*
Muscle soreness (0–6)	3.75 (0.34)	3.99 (0.42)	0.24 (−1.13 to 1.61)	3.73 (0.34)	−0.02 (−1.21 to 1.18)	0.68 (0.30)	−0.07 (−1.16 to 1.02)	3.56 (0.29)	−0.19 (−1.24 to 0.87)
Sleep (h)	8.02 (0.24)	8.06 (0.29)	0.04 (−0.88 to 0.97)	7.53 (0.24)	−0.49 (−1.29 to 0.32)	7.75 (0.21)	−0.26 (−0.99 to 0.47)	7.69 (0.21)	−0.33 (−1.04 to 0.38)
Total wellness	18.39 (1.25)	18.14 (1.42)	−0.26 (−3.94 to 3.43)	17.09 (1.25)	−1.31 (−4.55 to 1.94)	16.54 (1.17)	−1.85 (−4.79 to 1.09)	15.98 (1.15)	−2.41 (−5.25 to 0.43)*

*SEM, Standard error of measurement; MD, Mean difference, 95%CI, 95% confidence interval*.

* *p < 0.05*.

## Discussion

The aim of this study was to investigate the influence of COVID-19 on physical performance and mental health in professional football players. We retrospectively analyzed data from weekly assessments throughout the 2020–2021 season from the players of RAFC's first team. Our findings showed statistically significant differences in right leg eccentric Nordic hamstring strength, stress levels, mood scores and overall wellness, although the statistically significance was conversed by Bonferroni correction.

Results of physical performance outcomes were conflicting. Mean CMJ height improved at all time points after confirmed COVID-19, 2.45–4.32 cm, respectively. Also mean eccentric Nordic hamstring strength improved at all time points and was statistically significant at 2–4 weeks after the first positive PCR test prior to Bonferroni correction, namely 41.48 and 34.76 N. Although multiple comparisons counteraction corrected the possibility of making a type I error, these findings are still contradictory to physiological changes in athletes with viral diseases. Research has revealed that infection leads to decrease in muscle strength and endurance and enzyme activity as well as abnormal mitochondrial activity (Börjesson et al., 2017), resulting in loss of performance which can remain for several weeks after the infection (Roberts, 1986). The study of Grazioli et al. (2020) investigated whether parameters such as CMJ jump height and eccentric hamstring strength after a post SARS-CoV-2 infection quarantine were different than in a normal off-season period. Similar to our results, their results showed a significant increase in CMJ jump height. No significant differences were found in hamstring eccentric strength, however. Despite the improvements in CMJ height and eccentric Nordic hamstring strength, there was a reduction in mean isometric hip adduction and abduction strength for all time points after COVID-19 compared to 1 week prior, except mean abduction strength for the right side 8 weeks post SARS-CoV-2 infection. The tendency of adductor and abductor muscle strength results shows a cumulating performance reduction after two (MD: −9.13 to −21.31 N) and 4 weeks (MD: −16.89 to −33.40 N), to improve again after 6 weeks (MD: −3.75 to −18.46) and 8 weeks (MD: −10.14 to 2.05). This tendency could be explained by the period skeletal muscles need to replenish protein after infection (Börjesson et al., 2017). Specifically in COVID-19, myalgias are a common symptom, resulting from TMPRSS2-receptors which are targeted by the SARS-CoV-2 virus (Metzl et al., 2020). These myalgias reduce muscle performance but resolve after a few weeks (Metzl et al., 2020), which could possibly explain the tendency of adductor and abductor strength after COVID-19 contamination. In general, athletes are advised to not exercise during their infection due to possible aggravation of the infection and increased risk of injury as a result of impaired coordination and balance (Börjesson et al., 2017), which makes it rather difficult to tackle performance deficits. Therefore, it is advocated to initiate gradual return to play after the infection is resolved (Börjesson et al., 2017).

Results of the modified Hooper scale were unequivocal, showing reductions in all subscale scores at 1, 6, and 8 weeks post COVID-19 infection compared to the players' pre-infection scores. Prior to Bonferroni correction, reductions in players' mood scores, stress level scores and overall wellness scores were statistically significant 8 weeks post-infection, indicating more stress, a decreased mood and lower overall wellness on the long term. This is in accordance with recent COVID-19 related research. A recent systematic review about the impact of the SARS-CoV-2 Coronavirus pandemic on professional athletes showed statistically significant deteriorations in mental health (Jurecka et al., 2021). Moreover, a study assessing both professional and non-professional football players in Spain found reduced sleep quality and mood states during the COVID-19 confinement (Mon-López et al., 2020). The results of this study also showed that decreased mood affected training variables and performance (Mon-López et al., 2020). Clinicians should take into consideration that social isolation due to the mandatory quarantine has considerable impact on mental health, and should therefore addressed appropriately (Mon-López et al., 2020).

## Strengths And Limitations

Although our total sample consisted of 33 players, only 11 players were included in the data analysis. Then again, this means that 33% of the players was infected with SARS-CoV-2. The small sample size in this study yielded large CI's and thus less precise estimates of effects (Higgins and Cochrane Collaboration, 2020). Furthermore, multiple hypotheses were tested, increasing the likelihood for type I errors (Armstrong, 2014). Therefore Bonferroni correction was applied. However, this method could increase the likelihood for type II errors (Armstrong, 2014). Therefore, our results are described both prior and post Bonferroni correction, to highlight the clinically relevant results. This study was a retrospective cohort study of the database from the pre-season testing and weekly assessments throughout the season. Retrospective cohort studies are prone to the occurrence of selection bias (Powell and Sweeting, 2015). However, the selected cohort in the current study is representative for professional football players with COVID-19 contamination and therefore minimized selection bias (Sedgwick, 2014). Another possible disadvantage of retrospective studies is recall bias, typically present in studies when there are selective presumptions regarding the study outcomes (Sedgwick, 2014). RAFC, like many professional sports organizations, performs customary pre-season testing and weekly assessments to be able to compile physical profiles of the club's players (Ryan et al., 2019). These assessments were not specifically performed to inquire about the effect on COVID-19 contamination. Hence, recall bias is minimized. Due to the retrospective nature of this study, we were unable to include a control group. Hence, we could not assess the possible involvement of a detraining effect. Due to the recent COVID-19 pandemic, a lot of research is surfacing regarding the effects of the virus on sports performance. To the best of our knowledge, there is no existing research regarding the direct effect of COVID-19 contamination on physical performance and mental health during the season, however. All outcomes were assessed with reliable and valid measurement materials and methods, in standardized circumstances. Although the results of this study are inconclusive, our findings could give relevant information for management of similar sports clubs. Furthermore, these results could be the foundation of a rationale regarding a large-scale prospective study researching the effect of COVID-19 infection on physical performance and mental health.

## Conclusion

This study retrospectively investigated whether physical performance and mental well-being would change after COVID-19. The effect on physical performance is conflicting, indicating an increase in CMJ height and an improvement in eccentric Nordic hamstring strength whereas mean isometric hip adduction and abduction strength reduced. Mental wellness scores deteriorated in time after a confirmed SARS-CoV-2 infection. For medical professionals and physical performance trainers to have a better understanding of how SARS-CoV-2 infection influences athletes, future prospective research is advocated to evaluate the effect of COVID-19 on physical performance and mental health in professional athletes.

## Data Availability Statement

The data analyzed in this study is subject to the following licenses/restrictions: Dataset is kept privately in the club because data from professional athletes were analyzed. Requests to access these datasets should be directed to jente.wagemans@uantwerpen.be.

## Ethics Statement

Ethical review and approval was not required for the study on human participants in accordance with the local legislation and institutional requirements. The patients/participants provided their written informed consent to participate in this study.

## Author Contributions

JW: conceptualization, validation, formal analysis, and writing—original draft. PC and JV: conceptualization, investigation, validation, methodology, and writing—review and editing. JJ, XC, and CC: data curation and investigation. DV: conceptualization, writing—review and editing, and supervision. All authors contributed to the article and approved the submitted version.

## Conflict of Interest

The authors declare that the research was conducted in the absence of any commercial or financial relationships that could be construed as a potential conflict of interest.

## Publisher's Note

All claims expressed in this article are solely those of the authors and do not necessarily represent those of their affiliated organizations, or those of the publisher, the editors and the reviewers. Any product that may be evaluated in this article, or claim that may be made by its manufacturer, is not guaranteed or endorsed by the publisher.
